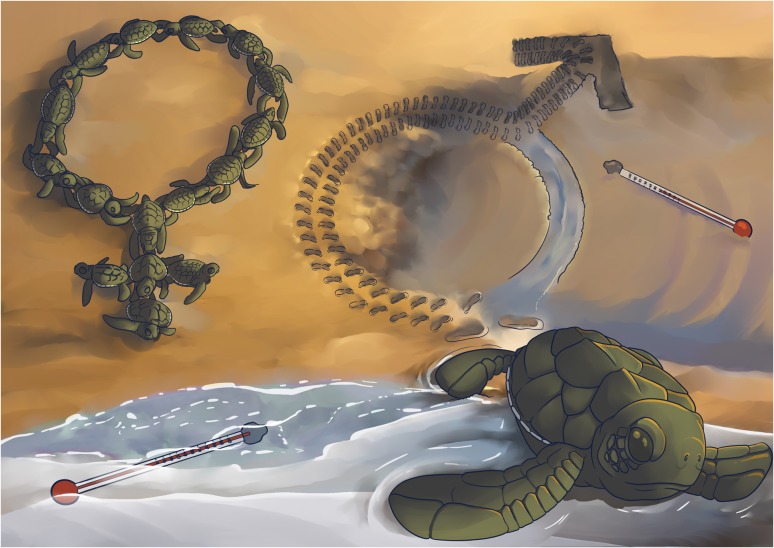# The future is female. Is that a problem for sea turtle conservation?

**DOI:** 10.1093/conphys/coy009

**Published:** 2018-03-01

**Authors:** Lisa M Komoroske

**Affiliations:** Department of Environmental Conservation, University of Massachusetts Amherst, 160 Holdsworth Way, Amherst, MA 01002, USA

Ask any biologist, and they will tell you: Sex is important. One of the key reasons it is important is that, for many species, sustaining healthy populations requires enough females and males that can mate and reproduce. This is why a new study determining that one of the largest green sea turtle nesting grounds in the world has been producing almost all females for over two decades is so troubling.

Unlike humans, sea turtle sex is determined by environmental temperatures—warmer nests produce more females, and cooler nests produce more males. So, biologists have long been concerned about potential looming effects of warming sand temperatures on sea turtle sex ratios (how many females to males in a population). But how could scientists study this? Sea turtle sex ratios can be hard to quantify: young males and females look essentially the same, take years to reach maturity, and travel thousands of kilometres between feeding and nesting beaches. Internally, sea turtles have different reproductive organs, but as IUCN-listed species, sacrificing turtles to ‘look under the hood’ is not allowed. For years, these limitations tied the hands of researchers, despite growing urgency. Enter the fields of Conservation Physiology and Genetics.

Biologists Jensen et al. (2018) developed a novel, non-lethal method by combining tools from endocrinology and population genetics. They applied this approach at key green turtle feeding grounds in Australia. First, they took a blood sample and measured testosterone to determine if the turtle was male or female. At the same time, they extracted DNA to determine the turtle’s origin. Because turtles return to reproduce in the same regions where they hatched, turtles from northern Great Barrier Reef (nGBR) beaches have distinct genetic signatures from those from southern (sGBR) beaches. Putting these pieces together, the researchers determined that turtles from nGBR beaches were almost exclusively females! While female-bias is common, what they found was extreme: over 99% of juveniles and sub-adults were female. This contrasted with older nGBR turtles and animals from cooler sGBR beaches. These findings, combined with warming sand temperature data over the last several decades, point to climate change as the likely cause. This is the first study to demonstrate such sublethal impacts of climate change, with strong potential to shape sea turtle populations in coming decades.

Where do we go from here? This knowledge could help conservation biologists develop approaches to mitigate warming, like shading nests to reduce incubation temperatures. Fully understanding long-term implications will require more research. How common are these patterns in other sea turtle populations and species? And, for long-term viability of sea turtle populations with strong female-biases, how many males are enough?

From a broader perspective, this study adds to mounting evidence that human-caused climate change has already profoundly impacted wildlife populations around the globe. Our continued efforts and long-term commitments are needed to drastically reduce carbon emissions to mitigate climate change impacts. Perhaps, this illustration of such an iconic species can push us to redouble our own efforts.

Illustration by Erin Walsh; Email: ewalsh.sci@gmail.com

**Figure:**